# Fabrication and Characterization of Biocompatible Multilayered Elastomer Hybrid with Enhanced Water Permeation Resistance for Packaging of Implantable Biomedical Devices

**DOI:** 10.3390/mi15111309

**Published:** 2024-10-28

**Authors:** Dae Hyeok An, Hee Cheol Kang, Jun Woo Lim, Junho Kim, Hojin Lee, Jae Hyun Jeong, Sung-Min Park, Jae Woo Chung

**Affiliations:** 1 Department of Materials Science and Engineering, Soongsil University, 369 Sangdo-ro, Dongjak-gu, Seoul 06978, Republic of Korea; shavros@gmail.com (D.H.A.); wkdehrgml@gmail.com (H.C.K.); 2 Department of Chemical Engineering, Soongsil University, 369 Sangdo-ro, Dongjak-gu, Seoul 06978, Republic of Korea; ljw9424@soongsil.ac.kr (J.W.L.); nfejjh@ssu.ac.kr (J.H.J.); 3 Department of Convergence IT Engineering, Pohang University of Science and Technology (POSTECH), Pohang 37673, Republic of Korea; iwog3927@postech.ac.kr (J.K.); sungminpark@postech.ac.kr (S.-M.P.); 4 Department of Intelligent Semiconductors, Soongsil University, 369 Sangdo-ro, Dongjak-gu, Seoul 06978, Republic of Korea; hojinl@ssu.ac.kr

**Keywords:** cytocompatibility, hexadecyl-modified SiO_2_ (HD-SiO_2_), implantable biomedical device package, polydimethylsiloxane (PDMS), polydopamine (PDA)

## Abstract

This study presents the synthesis and characterization of hexadecyl-modified SiO_2_ (HD-SiO_2_) nanoparticles and their application in the fabrication of a multilayered elastomer hybrid sheet to enhance water resistance in implantable biomedical devices. The surface modification of SiO_2_ nanoparticles was confirmed via FT-IR and TGA analyses, showing the successful grafting of hydrophobic hexadecyl groups. FE-SEM and DLS analyses revealed spherical HD-SiO_2_ nanoparticles with an average size of 360 nm. A multilayered elastomer hybrid sheet, consisting of a PDMS-based circuit-protecting body, a water resistance layer of HD-SiO_2_, a planarization layer, and a biocompatible layer of polydopamine, was fabricated and characterized. The water resistance layer exhibited superhydrophobic properties, with a water contact angle of 154.7° and a 27% reduction in water vapor transmission rate (WVTR) compared to the circuit-protecting body alone. The device packaged with both the circuit-protecting body and water resistance layer demonstrated a tenfold increase in operational lifespan in water medium compared to the device without the water resistance layer. Cytotoxicity and cell proliferation tests on human dermal fibroblast cells (HDFn) confirmed the biocompatibility of the multilayered sheet, with no significant cytotoxicity observed over 48 h.

## 1. Introduction

The rise in ultra-aging populations and improved living standards have led to increased interest in biomedical devices that use advanced engineering and life-science technologies [1,2]. Implantable devices, such as neurostimulators, electrocardiograms, and blood glucose sensors, are particularly noted for their potential to provide more accurate and comfortable healthcare [3,4]. These devices are now seen as essential for extending life and enhancing the quality of life in modern society. For these devices to be successfully implanted, they need to function reliably in the human body’s fluids and electrolytes, and avoid causing any adverse reactions [5,6]. Thus, selecting the suitable exterior packaging materials to protect these devices is very important. Although metals like titanium are commonly used for device packaging, these come with challenges, such as high cost, complicated processing, and lack of flexibility, which can be problematic inside the human body [7,8]. As a result, there is increasing research into using polymers for packaging implantable biomedical devices because they are more cost-effective and flexible, and easier to manufacture.

Among the many polymers being studied, poly (dimethyl siloxane) (PDMS) was broadly applied in biomedical devices such as artificial skin and organs because of its adjustable hardness, convenient molding, good optical transparency, and nontoxic properties [9]. However, PDMS is an intrinsically hydrophobic and bioinert material, which limits its applications in implantable devices [10,11]. Furthermore, due to its inherent free volume, PDMS has relatively high gas and moisture diffusion and permeability compared to metals [12]. These characteristics make it challenging to protect internal electronic circuits or batteries from biological moisture. Thus, there is a critical need to develop advanced PDMS-based packaging materials for implantable biomedical devices that both reduce water permeation and improve biocompatibility. Most studies into improving the moisture resistance of implantable devices focus on the incorporation of nanomaterials such as SiO_2_, cellulose nanocrystals (CNC), graphene, and MnO into packaging materials [13,14,15]. This approach has the advantage of being relatively simple to use in manufacturing; however, it has limitations in improving moisture resistance. Additionally, atomic layer deposition (ALD) is an effective way to prevent the penetration of moisture into the packaging [16]. However, the ALD process involves high costs, long processing times, and challenges in large-scale production. Thus, it is necessary to develop an effective strategy to concurrently improve both the water barrier properties and biocompatibility of the device package.

In this study, we developed a multilayered elastomer hybrid sheet for use as a packaging material in implantable biomedical devices. The multilayered elastomer hybrid sheet consists of a circuit-protecting body and three functional layers: a hydrophobic silica nanoparticle-based water resistance layer, a PDMS planarization layer, and a biocompatible polydopamine (PDA) layer. Our results demonstrate that the water resistance layer effectively reduces water permeation, while the PDA layer enhances biocompatibility. Consequently, the multilayered elastomer hybrid sheet shows significant potential as a soft packaging material for implantable biomedical devices, offering stable performance over extended periods within the body.

## 2. Materials and Methods

### 2.1. Materials

Poly (dimethyl siloxane) (PDMS, Sylgard 184) and the corresponding curing agent were supplied by Dow Corning (Midland, MI, USA). Tetraethyl orthosilicate (TEOS, 98%), hexadecyltrimethoxysilane (HDTMS, ≥85%), and dopamine hydrochloride were purchased from Sigma Aldrich, Yongin-si, Republic of Korea. Tetrahydrofuran (THF, ≥99.5%) and ethyl alcohol (EtOH, ≥95%) were purchased from Daejung Chemical and Metals Co., Ltd., Siheung-si, Republic of Korea. Ammonia solution (NH_4_OH, 28%) and Tris-HCL buffer solution (pH 8.5, 1 M) were supplied by JUNSEI Co., Ltd., Tokyo, Japan and T&I biotechnology, respectively. All the chemicals were used as received without further purification.

### 2.2. Preparation of the Hexadecyl-Modified Silica Nanoparticles (HD-SiO_2_) and the HD-SiO_2_ Coating Solution

Initially, 3 mL of NH_4_OH was mixed with 50 mL of ethanol in a 100 mL 3-neck round-bottom flask to form a homogeneous solution, stirring continuously for 30 min at 50 °C under a nitrogen atmosphere. Afterward, 3 mL of tetraethyl orthosilicate (TEOS) was added dropwise while stirring continuously, using a dropping funnel to control the addition interval. The reaction continued for 2 h, forming a transparent silica nanoparticle. After that, 1% HDTMS was added to the nanoparticle solution to modify the hydrophilic hydroxyl surface of silica nanoparticles into a hydrophobic alkyl surface. The reaction was allowed to continue for a further 2 h, with continued stirring at 50 °C to form hexadecyl-modified silica nanoparticles (HD-SiO_2_). The HD-SiO_2_ nanoparticles were then collected by centrifugation at 10,000 rpm for 20 min and re-dispersed in ethanol. After three cycles of centrifugation and re-dispersion, the HD-SiO_2_ nanoparticles were vacuum-dried overnight. To form the HD-SiO_2_ nanoparticle coating solution, PDMS (0.55 g) and the corresponding curing agent (0.055 g) were mixed with THF (55 mL) by magnetic stirring for 1 h at room temperature. The HD-SiO_2_ (with HD-SiO_2_/PDMS mass ratio of 2:1) was then dispersed in the solution with the aid of ultrasonication for 30 min.

### 2.3. Fabrication of the Multilayered Elastomer Hybrid Sheet

**Figure 1:**
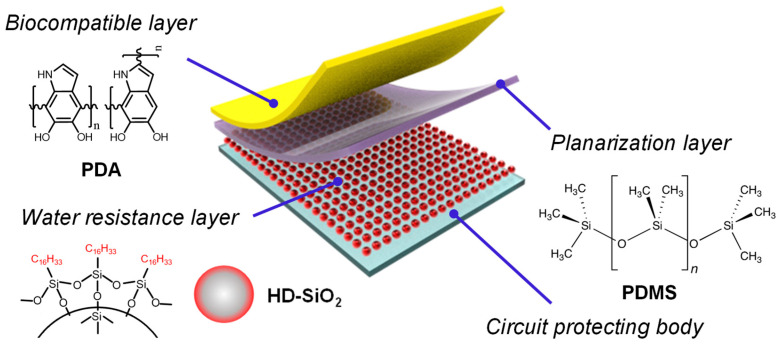
Schematic structure of the multilayered elastomer hybrid sheet composed of a circuit-protecting body, a water resistance layer, a planarization layer, and a biocompatible layer.

PDMS was mixed thoroughly with the corresponding curing agent in a 10:1 ratio by weight and degassed under vacuum for 1 h. The mixture was poured into a mold (20 × 20 × 1.0 mm^3^) and cured at 100 °C for 15 min using a hot press. After being cooled to room temperature, the silicon elastomer sheet (also called the circuit-protecting body) was removed from the mold, and the HD-SiO_2_ coating solution was then sprayed onto the surface of the silicon elastomer sheet using an airbrush with a 0.5 mm nozzle (ATOMAX, am6s-0.5) to form the water resistance layer on the surface of the circuit-protecting body. Operating air was controlled with the airbrush compressor at 25 psi, and the distance between the airbrush and the substrate was kept at 5 cm depending on the coating area. During the spray coating, the airbrush was moved smoothly to ensure the uniform coverage of the coating. After spray coating, the sheet was dried in the air for 24 h to remove the residual solvent, resulting in the formation of the water resistance layer composed of the HD-SiO_2_. After the formation of the water resistance layer on the top of the circuit-protecting body, the PDMS/curing agent mixture was applied to the top of the water resistance layer through spray coating, similar to the method mentioned above, in order to form the planarization layer. This multilayered elastomer sheet was immersed in a freshly prepared 0.2 mg mL^−1^ dopamine hydrochloride aqueous solution with pH 8.2 in an open beaker and was stirred using an orbital shaker in a dark environment to form the biocompatible layer on the surface of the planarization layer through the deposition of polydopamine (PDA). Non-firmly adsorbed PDA particles were washed with ethanol and deionized water and dried in a vacuum oven at room temperature for 24 h, resulting in the multilayered elastomer hybrid sheet composed of a circuit-protecting body, a water resistance layer, a planarization layer, and a biocompatible layer, as shown in Figure 1.

### 2.4. Characterization

The attenuated total reflection Fourier-transform infrared (ATR FT-IR) spectra were recorded on a Bruker Vertex 70 spectrometer (Billerica, MA, USA) with a spectral resolution of 2 cm^−1^ over the range of 4000–600 cm^−1^ to confirm the synthesis of the HD-SiO_2_ nanoparticles and the fabrication of the multilayered elastomer hybrid sheet. To estimate alkyl chain content in the HD-SiO_2_ nanoparticles, thermal degradation curves were obtained using a Mettler Toledo TGA/DSC1 thermogravimetric analyzer (TGA). The thermogravimetric curves were recorded in the temperature range of 30–700 °C under nitrogen at a heating rate of 10 °C min^−1^. The morphology of HD-SiO_2_ nanoparticles and the surface and cross-sectional morphologies of the multilayered elastomer hybrid sheet were observed by field-emission scanning electron microscopy (FE-SEM, Carl Zeiss GeminiSEM 300, Jena, Germany) with energy-dispersive X-ray spectroscopy (EDXS, BRUKER XFlash 6-30, Billerica, MA, USA). The FE-SEM samples were coated with a thin conductive Pt layer prior to observation. Diameters of HD-SiO_2_ nanoparticles suspended in THF were determined using dynamic light scattering (DLS, Otsuka Electronics DLS-8000 HL, Osaka, Japan) with a 90° scattering angle. The measured time correlation functions were analyzed by autocorrelation using the method of cumulants. The WVTR (water vapor transmission rate) values were measured using a Permatran-W 3/33 MA instrument (Mocon, Minneapolis, MN, USA) at 38 ± 2 °C and 100% relative humidity for 24 h, according to ASTM F1249. The dimensions of the sample for the WVTR measurements were 40 (L) × 40 (W) × 0.5 (D) mm^3^. The water contact angle was evaluated using a DSA 100 instrument (Krüss, Hamburg, Germany). A 4 µL portion of distilled water was dropped five times at different positions on the film and the average value was calculated.

### 2.5. Lifetime Test in Water Medium

To evaluate the water resistance of the multilayered hybrid sheets, we first prepared a board capable of measuring temperature and humidity. This board was designed to operate only when power was supplied wirelessly from an external source, minimizing environmental changes caused by its operation. When the WPT TX coil was close to the RX coil on the board, power was supplied to the board, and data were transmitted to a receiver connected to a PC. Once the MCU was powered on, it communicated with the temperature and humidity sensor (BME280, Bosch Sensortec, Reutlingen, Germany) via SPI to read the current temperature and humidity data. The temperature could be measured within a range of −40 °C to +85 °C with a 20-bit resolution, and the humidity could be measured within a range of 0 to 100% relative humidity with a 16-bit resolution. The raw data were transmitted to the receiver via 2.4 GHz wireless communication, where they were converted into temperature and humidity values on the PC and recorded. Then, two types of devices were fabricated: one with only the circuit-protecting body as packaging for the designed board, and the other with both the circuit-protecting body and the water resistance layer. The fabricated devices were subjected to accelerated conditions by immersing them in a thermostatic water bath at 60 °C. Signal reception was measured every 12 h to analyze their lifespan.

### 2.6. Cytotoxicity and Cell Proliferation Tests

The in vitro biocompatibility of the multilayered elastomer hybrid sheet was evaluated using human dermal fibroblasts (HDFn, Gibco, Waltham, MA, USA). HDFn cells were cultured in a growth medium consisting of Medium 106 (M106, Gibco), 10% Low Serum Growth Supplement (LSGS, Gibco), and 1% penicillin-streptomycin (P/S, 100×, Biowest). The cells (5.0 × 10^3^ cells/well) were seeded in 96-well plates containing various submerged substrates (2.0 mg each: biocompatible layer, water-resistant layer, and circuit-protecting body) for 6, 12, and 48 h. Following this, the cells were incubated in a proliferation medium containing 0.5 mg/mL of MTT. After a 4 h incubation, a lysis buffer solution (50% (*w*/*v*) N,N-Dimethylformamide (DMF) in distilled water, and 10% (*w*/*v*) sodium dodecyl sulfate (SDS)) was added to each well to terminate the reaction. The 96-well plates were then incubated at room temperature for 2 h to allow the diffusion of formazan into the medium. Absorbance was measured at 570 nm using a microplate reader (Thermo Fisher Scientific, Waltham, MA, USA). The optical densities (ODs) at 0, 6, 12, 24, and 48 h after the assays were converted to relative cell viability (%) by normalizing to the 0 h and control group values.

To assess the cell proliferation potential and biocompatibility of each substrate of the multilayered elastomer hybrid sheet, HDFn cells were seeded on the surface of a cell culture plate (control) and various substrates of X (the biocompatible layer, water-resistant layer, and circuit-protecting body) with dimensions of 1.0 × 1.0 cm^2^ at a concentration of 1 × 10⁵ cells/cm^2^. The cells were submerged in a culture medium and incubated for 48 h at 37 °C and 5% CO_2_ in the proliferation medium. Cell morphology and density were observed and captured using a microscope (Nikon, Tokyo, Japan) at 0, 6, 12, 24, and 48 h.

## 3. Results and Discussion

### 3.1. Synthesis of HD-SiO_2_

**Figure 2:**
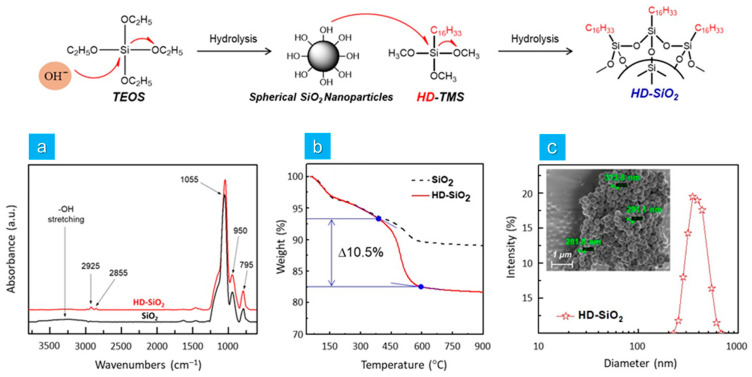
Schematic representation of the synthesis process and characterization of SiO_2_ and HD-SiO_2_. (**a**) FT-IR spectra and (**b**) TGA thermograms of SiO_2_ and HD-SiO_2_ nanoparticles. (**c**) DLS plot of HD-SiO_2_ nanoparticles. (Inset: FE-SEM image of HD-SiO_2._).

The synthesis and surface modification of SiO_2_ nanoparticles were characterized using FT-IR spectroscopy. Figure 2a presents the FT-IR spectra of both pristine SiO_2_ and hexadecyl-modified SiO_2_ (HD-SiO_2_) nanoparticles. The neat SiO_2_ nanoparticles exhibited two prominent absorption peaks at 1055 cm^−1^ and 795 cm^−1^, corresponding to the stretching vibrations of Si-O-Si, along with a peak at 950 cm^−1^, indicative of Si-OH stretching vibrations [17]. Furthermore, a broad absorption band in the range of 3000–3500 cm^−1^ was detected, attributed to the presence of numerous hydroxyl groups on the surface of the SiO_2_.

The HD-SiO_2_ nanoparticles also exhibited absorption peaks corresponding to the stretching vibrations of Si-O-Si and Si-OH at 1055, 795, and 950 cm^−1^, respectively. However, two additional peaks were observed in the FT-IR spectrum of HD-SiO_2_ at 2925 and 2855 cm^−1^, corresponding to the asymmetric and symmetric stretching of the CH_2_ group. Notably, the surface hydroxyl absorption peak was significantly reduced in the FT-IR spectrum of HD-SiO_2_ [18], indicating the successful synthesis of the silica nanoparticles and effective grafting of hydrophobic hexadecyl groups onto their surface. The presence of these hexadecyl groups on the HD-SiO_2_ nanoparticles was further confirmed by TGA analysis. As shown in Figure 2b, both SiO_2_ and HD-SiO_2_ nanoparticles exhibited an initial weight loss in the temperature range of 75–150 °C, corresponding to the release of adsorbed water from their surfaces. Additionally, both nanoparticles demonstrated a second weight loss beyond 350 °C. For the SiO_2_ nanoparticles, this second weight loss of approximately 3 wt% occurred between 450 and 600 °C, which is attributed to the thermal decomposition of hydroxyl moieties on the SiO_2_ surface [19]. In contrast, the HD-SiO_2_ nanoparticles exhibited a more significant second weight loss of approximately 10.5 wt% in the temperature range of 390–600 °C, which can be attributed to the decomposition of the hexadecyl groups attached to the HD-SiO_2_ nanoparticles. This observation was in good agreement with the FT-IR results. Figure 2c shows that the HD-SiO_2_ nanoparticles were spherical in morphology with a narrow size distribution, and the average size determined by DLS was approximately 360 nm in diameter. Therefore, the combined results from FT-IR, TGA, SEM, and DLS confirmed the successful synthesis of spherical HD-SiO_2_ nanoparticles with hydrophobic hexadecyl groups on their surface.

### 3.2. Manufacture of the Multilayered Elastomer Hybrid Sheet

**Figure 3:**
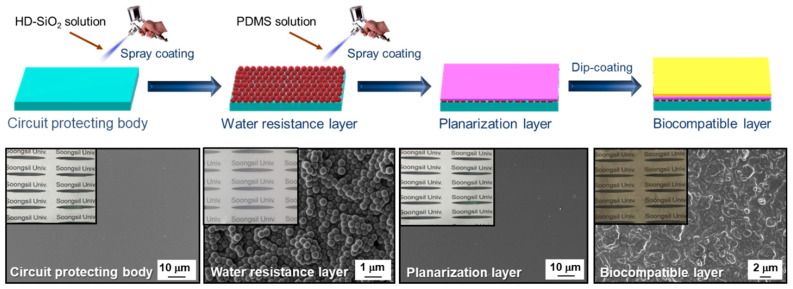
The FE-SEM images of the surface of the multilayered elastomer hybrid sheet according to its manufacturing steps. (Inset: the photo images of the surface of the multilayered elastomer hybrid sheet at each manufacturing step).

The surface morphology of the multilayered elastomer hybrid sheet was systematically analyzed at each stage of the manufacturing process using FE-SEM. As shown in Figure 3, the PDMS-based circuit-protecting body initially exhibited a transparent and smooth surface. Upon spray coating of HD-SiO_2_ onto the PDMS surface, a uniform deposition of HD-SiO_2_ nanoparticles was observed, confirming the successful formation of the water resistance layer on the circuit-protecting body substrate. Subsequently, the application of the planarization layer, achieved by coating the water resistance layer with PDMS, restored a smooth surface texture. Finally, the deposition of PDA on the planarization layer to form the biocompatible layer resulted in a notably rough surface morphology. This roughness is formed by the self-polymerization of dopamine, which occurs in weakly basic conditions, causing micro-scale aggregations [20].

**Figure 4:**
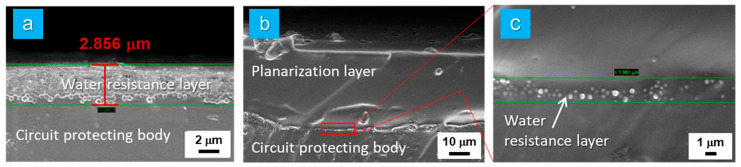
(**a**) The fracture surface image observed by FE-SEM after the spray coating of HD-SiO_2_ onto the surface of the PDMS-based circuit-protecting body. (**b**) The fracture surface image observed by FE-SEM after the formation of the planarization layer on the top of the water resistance layer. (**c**) The magnified fracture surface image of Figure 4b.

In particular, as depicted in Figure 4a, the water resistance layer composed of HD-SiO_2_ was well established. After the HD-SiO_2_ spray coating, this layer exhibited a thickness of approximately 2.856 µm on the surface of the PDMS-based circuit-protecting body.

Moreover, Figure 4b,c demonstrate that the water resistance layer was precisely located between the circuit-protecting body and the planarization layer in a partially laminated form, with no structural degradation observed even after the application of the planarization layer. These observations provide clear evidence of the successful fabrication of a multilayered elastomer hybrid sheet, consisting of the circuit-protecting body, water resistance layer, planarization layer, and biocompatible layer.

**Figure 5:**
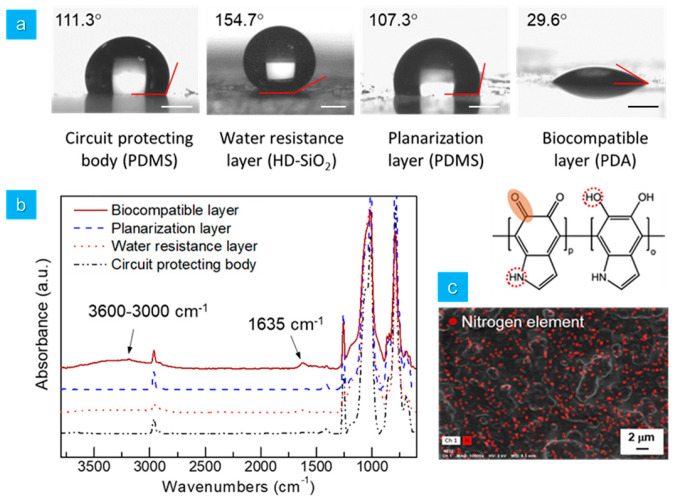
(**a**) Water contact angle images at various stages of the manufacturing process. White and black scale bar = 1 mm. (**b**) FT-IR spectra corresponding to each fabrication stage. (**c**) FE-SEM EDX mapping image showing the nitrogen elemental distribution within the biocompatible PDA layer.

Water contact angle measurements further substantiated the stable formation of the multilayered elastomer hybrid sheet. As shown in Figure 5a, the PDMS-based circuit-protecting body exhibited a water contact angle of approximately 111.3°, while the water resistant layer demonstrated superhydrophobic properties with a contact angle of around 154.7°, suggesting its efficacy in preventing moisture penetration within the multilayered elastomer hybrid sheet. In fact, the WVTR value for the circuit-protecting body was approximately 59.3 g m^−1^ day^−1^; however, when the water-resistant layer was applied using HD-SiO_2_, the WVTR value decreased to 43.5 g m^−1^ day^−1^, indicating a reduction in moisture permeability of approximately 27% compared to the circuit-protecting body alone. The planarization layer restored the characteristic water contact angle of PDMS, and the biocompatible PDA layer exhibited hydrophilic properties, with a water contact angle of approximately 29.6°. As shown in Figure 5b, a broad peak corresponding to the stretching of hydroxyl and amine groups of the biocompatible PDA layer was observed within the range of 3000 to 3600 cm^−1^, and the conjugated carbonyl peak of PDA was distinctly identified at 1635 cm^−1^ [21]. Furthermore, elemental analysis of the biocompatible layer surface using energy-dispersive X-ray spectroscopy (EDX) mapping revealed a uniform distribution of nitrogen across the biocompatible layer, confirming the successful formation of the hydrophilic and biocompatible PDA layer on the outermost surface of the multilayered elastomer hybrid sheet.

Thus, based on the combined results from FE-SEM, water contact angle measurements, FT-IR, and EDX, it is evident that the multilayered elastomer hybrid sheet, composed of the circuit-protecting body, water resistant layer, planarization layer, and biocompatible layer, was successfully fabricated.

### 3.3. A Lifetime Test of the Multilayered Elastomer Hybrid Sheet in a Water Medium

**Figure 6:**
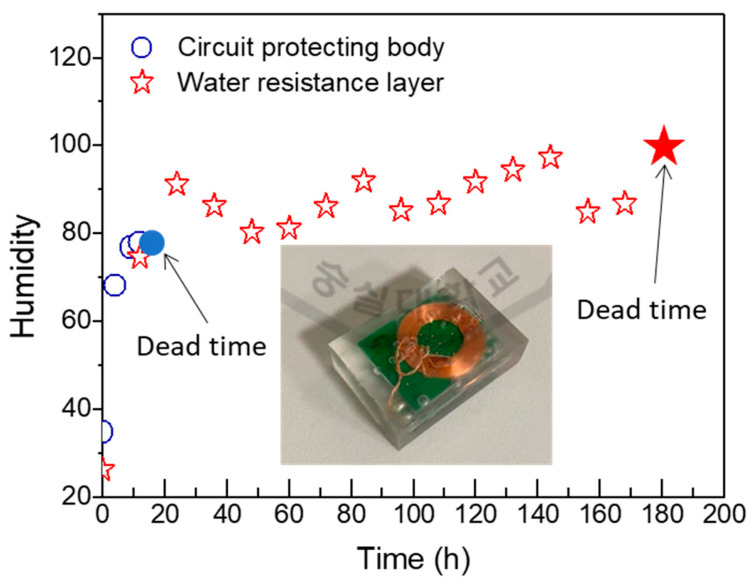
The lifetime test of the circuit device packaged with only the circuit-protecting body and packaged with both the circuit-protecting body and the water resistance layer.

A lifetime test was conducted to assess the water resistance of the circuit device, comparing those packaged with only the PDMS-based circuit-protecting body and those with both the circuit-protecting body and the water resistance layer. The test was carried out in a water bath at 60 °C. If water molecules penetrated the packaging and reached the interior, the device was able to measure and transmit real-time temperature and humidity data to a receiver. However, when an excessive number of water molecules infiltrated, the device could no longer measure humidity, resulting in a failure to transmit data to the receiver. As shown in Figure 6, the circuit device packaged solely with a circuit-protecting body failed to operate after 16 h of humidity exposure in a thermostatic water bath. However, the circuit device packaged with both the circuit-protecting body and water resistance layer remained operational for 180 h, over 10 times longer than the device with only the circuit-protecting body. These results indicate that the water resistance layer effectively prevented moisture penetration into the packaging, which was consistent with the previously observed WVTR results.

Based on these results, the Arrhenius equation was applied to predict the lifespan of the circuit devices at physiological temperature as follows [14,22],

(1) $$\frac{t_{2}}{t_{1}}=exp\;\left[\frac{E_{a}}{R}\left(\frac{1}{T_{2}}-\frac{1}{T_{1}}\right)\right]$$

where *R* is the gas constant; *T*_1_ and *t*_1_ are the temperature and the lifetime, respectively, under normal conditions; *T*_2_ and *t*_2_ are the temperature and the lifetime, respectively, under accelerated conditions; and *E_a_* is the activation energy. The activation energy for water diffusion in PDMS was reported to be approximately 14 kJ/mol [23], and *t*_1_ and *t*_2_ represent the lifetime during which the device can operate at the corresponding temperatures.

As a result, the circuit device packaged with both the circuit-protecting layer and the water resistance layer was estimated to operate for approximately 439 h under physiological conditions. In contrast, the device packaged with only the circuit-protecting body exhibited an estimated lifespan of around 39 h. This indicates that the addition of the water resistance layer extended the device’s operational lifespan by a factor of approximately 11.3.

### 3.4. Cytotoxicity and Cell Proliferation Tests of the Multilayered Elastomer Hybrid Sheet

**Figure 7:**
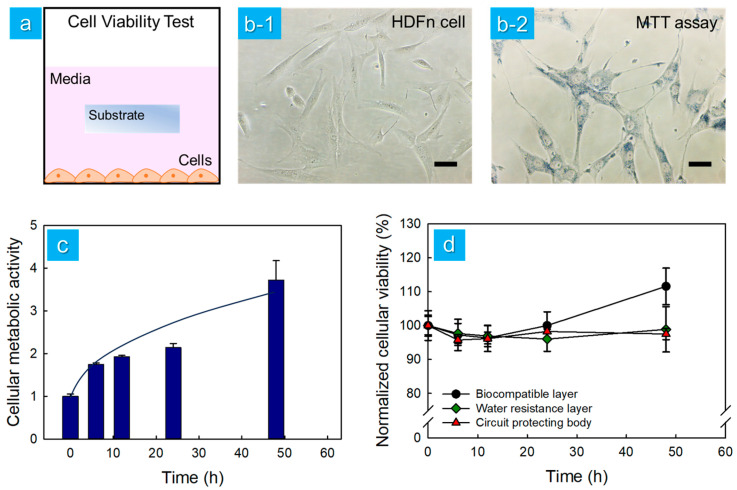
Cytotoxicity characterization of human dermal fibroblasts (HDFn) cells by each substrate of the multilayered elastomer hybrid sheet. (**a**) Schematic design of the cell viability test. (**b**) Bright field images of HDFn on a culture plate (**b-1**) and results from the MTT assay for cytotoxicity (**b-2**). (Scale bar: 100 μm.) (**c**) The cellular metabolic activity measured at each time point was normalized to the metabolic activity recorded immediately after cell stabilization in the well. (**d**) The cell viability measured at each time point was normalized to the values of the pure medium group at the corresponding time, comparing the biocompatible layer, the water resistance layer, and the circuit-protecting body.

The cytotoxicity of human dermal fibroblast (HDFn) cells was assessed on each substrate of the multilayered elastomer hybrid sheet. For the cell viability analysis, each substrate was standardized to a size of 2 × 2 mm, with the weight of the standardized substrates being approximately 2 mg. HDFn cells at a concentration of 5.0 × 10⁴ cells/mL were cultured with submerged substrates (2 × 2 mm^2^, 2 mg) in 96-well plates (Figure 7a). To evaluate cell viability, a qualitative LIVE/DEAD stain was performed along with the MTT assays. The images showed live cells, which matched the quantitative data from the MTT assay (Figure 7b-2). HDFn cells exhibited good proliferation, with a 3.7-fold increase in cellular metabolic activity at 48 h compared to 0 h (Figure 7c). As shown in the results of Figure 7c, cell viability increases over time due to cell proliferation. Therefore, cell viability at each time point should be normalized to that of the control group. By normalizing to the control group at each time point, it was observed that cells exposed to the biocompatible layer, the water resistance layer, and the circuit-protecting body showed no significant cytotoxicity up to 48 h, as shown in Figure 7d. The viability of HDFn cells remained statistically constant over the 48 h period (approximately 98%), confirming that all substrates comprising the multilayered elastomer hybrid sheet were non-cytotoxic.

**Figure 8:**
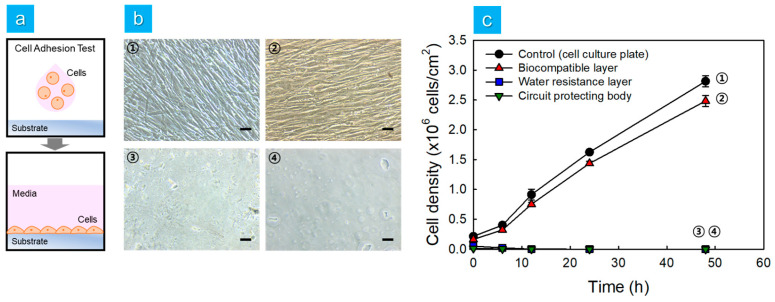
Adhesion and proliferation of HDFn cells on each substrate of the multilayered elastomer hybrid sheet. (**a**) Schematic design of the cell adhesion and proliferation test. (**b**) Bright field images of HDFn cells on various substrates: ① control (cell culture plate); ② biocompatible layer; ③ water resistance layer; ④ circuit-protecting body. (Scale bar: 100 μm) (**c**) Cell density of HDFn cells cultured on each substrate over time.

The adhesion and proliferation of HDFn cells were evaluated on three substrates (the biocompatible layer, the water resistance layer, and the circuit-protecting body) that comprise the multilayered elastomer hybrid sheet. After culturing HDFn cells in a 75-cm^2^ flask, they were seeded on the substrate surfaces at a concentration of 1 × 10⁵ cells/cm^2^. The substrates with cells were submerged and cultured in proliferation medium for 48 h (Figure 8a). The cells were observed and captured using a microscope at 0, 6, 12, 24, and 48 h. The number of cells per unit area was quantified from the images to calculate cell density. HDFn cells cultured on the water resistance layer and circuit-protecting body failed to adhere and proliferate. In contrast, cells adhered well to the biocompatible layer and formed a monolayer within 48 h (Figure 8b). The cell density of HDFn cells cultured on the biocompatible layer increased to approximately 2.48 × 10⁶ cells/cm^2^ at 48 h (Figure 8c). Notably, the cell density on the biocompatible layer was comparable to that observed on the cell culture plate (control). This demonstrated that incorporating a biocompatible layer into the multilayered elastomer hybrid sheet, which includes a circuit-protecting body and a water resistance layer, enhanced its suitability for application within the human body. In other words, this research illustrates the potential of multilayered elastomer hybrid sheets for use in packaging applications, such as neurostimulators and ECG (electrocardiogram) devices, which require cytocompatibility and water resistance for long-term use.

## 4. Conclusions

In this study, a multilayered elastomer hybrid sheet composed of a PDMS-based circuit-protecting body, a water resistance layer of HD-SiO_2_, a planarization layer, and a biocompatible layer of polydopamine was successfully fabricated. The water resistance layer significantly enhanced the moisture barrier properties, reducing water permeability by 27% and extending the operational lifetime of the packaged circuit devices over tenfold. Moreover, cytotoxicity and cell proliferation assays confirmed the biocompatibility of the multilayered elastomer hybrid sheet. This study demonstrates that the combination of HD-SiO_2_ and polydopamine effectively improves the water resistance and biocompatibility of polymeric packaging materials, offering a promising approach for the development of durable and stable implantable biomedical devices in physiological environments. This multilayered sheet was successfully developed for its intended purpose; however, interfacial adhesion remains a significant evaluation factor. Thus, future research to optimize the multilayer system will focus on evaluating interfacial adhesion and optimization related to layer thickness and number of layers.

## Data Availability

The original contributions presented in the study are included in the article. Further inquiries can be directed to the corresponding author.
